# Antibiofilm Activities of Tritrpticin Analogs Against Pathogenic *Pseudomonas aeruginosa* PA01 Strains

**DOI:** 10.3390/molecules30040826

**Published:** 2025-02-11

**Authors:** Gopal Ramamourthy, Hiroaki Ishida, Hans J. Vogel

**Affiliations:** Biochemistry Research Group, Department of Biological Sciences, University of Calgary, Calgary, AB T2N 1N4, Canada; rgopal@ucalgary.ca (G.R.); hishida@ucalgary.ca (H.I.)

**Keywords:** antibiofilm peptides, antimicrobial peptides, biofilms, diaminopropionic acid, *Pseudomonas aeruginosa*, tryptophan

## Abstract

In our previous work, we showed that short antimicrobial hexapeptides (AMPs) containing three Trp and three Arg residues had a potent antibiofilm activity against a pathogenic Gram-positive *Staphylococcus aureus* MRSA strain. However, the activity of these hexapeptides against a Gram-negative *Pseudomonas aeruginosa* PA01 strain was relatively poor. Herein, we tested the longer 13-residue synthetic AMP tritrpticin-NH_2_ (Tritrp) and several of its analogs as potential antibiofilm agents that can prevent biofilm formation (MBIC) and/or cause biofilm dissolution (MBEC) for two *P. aeruginosa* PA01 strains, one of which expressed the GFP protein. Tritrp, a porcine cathelicidin, is currently the only known naturally occurring cationic AMP that has three Trp in sequence (WWW), a feature that was found to be important in our previous study. Our results show that several Tritrp analogs were effective. In particular, analogs with Pro substitutions that had altered peptide backbone structures compared to the naturally occurring amphipathic two-turn structure showed more potent MBIC and MBEC antibiofilm activities. Selectivity of the peptides towards *P. aeruginosa* could be improved by introducing the non-proteinogenic amino acid 2,3-diaminopropionic acid, rather than Arg or Lys, as the positively charged residues. Using ^1^H NMR spectroscopy, we also reinvestigated the role of the two Pro residues in cis–trans isomerism of the peptide in aqueous solution. Overall, our results show that the WWW motif embedded in longer cationic AMPs has considerable potential to combat biofilm formation in pathogenic Gram-negative strains.

## 1. Introduction

Antibiotic resistance, where pathogenic bacterial strains can no longer be killed by commonly used antibiotics, is rapidly becoming an issue of widespread clinical concern [1,2,3]. In particular, pathogenic bacterial strains that have become resistant to multiple antibiotics are being detected with increasing frequency, particularly in hospital settings. This has led to the definition of the so-called ESKAPE pathogenic microorganisms, which require special attention, and many researchers are trying to explore new ways of combating infections caused by the six opportunistic disease-causing bacterial strains that make up this group [4,5,6].

Antimicrobial peptides (AMPs) offer a potential way of addressing the issue of antibiotic resistance. After the initial discovery of ribosomally-synthesized cationic AMPs in various animals in the 1980s, they have been shown to have the potential to kill or inhibit the growth of bacteria when they are in a free-living or ‘planktonic’ state [7,8]. They occur naturally in many organisms and have been found to attack bacteria in multiple ways, such as bacterial membrane disruption or destabilization, pore formation, or through targeting essential intracellular proteins, RNA/DNA synthesis, or by interfering in metabolic or regulatory pathways [9,10]. Numerous synthetic peptide analogs with improved killing characteristics and greater stability have now been tested against various disease-causing bacteria. Renewed interest in the clinical use of AMPs arose when it was found that they could also interfere in the formation of bacterial biofilms or cause the dissolution of preformed biofilms [11]. Nowadays, it is believed that such sessile communities of microbes, rather than planktonic bacteria, are responsible for more than two-thirds of all human infections [12]. Bacterial biofilms typically form on surfaces and they are usually made up of groups of cells that are encased in a matrix that is built-up of bacterially secreted polysaccharides, extracellular DNA, proteins and vesicles [13]. The matrix provides a protected environment that is difficult to penetrate for mammalian immune systems or for antibiotics. Consequently, much higher concentrations of antibiotics are required to treat bacteria that are living in biofilms compared to those existing in the free-living state [14,15]. Furthermore, biofilms also like to form on biomedical devices such as catheters and prosthetic joints, creating significant problems for clinicians [16]. In addition to bacteria, biofilms can also form for pathogenic fungi, such as *Candida albicans* (e.g., [17]). As such, the need has arisen for agents that can prevent the formation of biofilms of pathogenic microorganisms, or that can help in the breakdown of existing preformed biofilms. As a result, antibiofilm peptides are receiving considerable attention from researchers [14,18,19,20], although such peptides have not yet reached clinical application.

Pathogenic Gram-negative and Gram-positive strains, such as *Pseudomonas aeruginosa* and *Staphylococcus aureus,* often form biofilms and these two organisms have been studied frequently as representatives for the multidrug-resistant ESKAPE strains. However, the morphology and overall appearance of the biofilms made by these two organisms is quite distinct, and several studies have shown that they are sensitive to different concentrations of antibiofilm peptides [21]. Moreover, the process of biofilm formation and the efficacy of the subsequent attack by antibiofilm peptides further depends on the culture medium in which the cells are grown [21].

In two previous studies [22,23], we tested two different groups of antimicrobial peptides for their potency as antibiofilm agents acting on pathogenic *P. aeruginosa* PA01 and on *S. aureus* MRSA strains. Under the experimental conditions employed in those studies it was found that biofilms formed by the Gram-positive *S. aureus* strain were extremely sensitive to very low concentrations of Trp- and Arg-rich AMPs. In particular, hexapeptides made up of three Trp and three Arg residues could disrupt the *S. aureus* biofilms or prevent their formation, while being mostly ineffective against *P. aeruginosa*. Hexapeptides containing the three Trp in sequence (e.g., RRWWWR-NH_2_) proved to be the most effective and at higher concentrations, these could also act on *P. aeruginosa* [23]. These results prompted us to consider using longer Trp- and Arg-rich peptides as potential antibiofilm agents against the latter Gram-negative strain. For this purpose, we selected the 13-residue porcine cathelicidin peptide tritrpticin, which naturally contains the WWW sequence at its center and has an additional four Arg residues and a positively charged N-terminal end distributed around it (VRRFPWWWPFLRR). Tritrpticin was first described in 1996 [24] and has since been shown to have broad antimicrobial, antifungal as well as anticancer activities [25]. In an animal model study, its potential was shown for helping combat sepsis [26]. Tritrp is part of a group of potent, yet relatively short Trp- and Arg-rich AMPs; their shorter length makes them more economical to produce and hence they are an interesting target for further study [27,28]. Numerous variants of Tritrp, with natural and unnatural amino acid substitutions, have already been studied [29,30,31,32,33,34]. In most studies, it has been found that Tritrp acts as an antimicrobial on planktonic cells by perturbing bacterial membranes, although additional intracellular modes of action have not been excluded [31,34].

In this contribution, we used three distinct antibiofilm assays to study an amidated version of the Tritrp peptide and several analogs with selected amino acid substitutions. To our knowledge, this is the first time that the antibiofilm activity of Tritrp and its analogs have been surveyed. All the peptides used in this study were carboxy-amidated because the removal of the C-terminal negative charge improves the antibacterial activity, as is commonly seen for many AMPs [35]. The antimicrobial (MIC) and hemolytic activities of the majority of these peptides have been reported before, as well as their structural characterization by NMR spectroscopy [30,36,37]. The amino acid sequences of the Tritrp analogs used in this study, as well as those of the related Trp-rich bovine cathelicidin peptide indolicidin and the plant puroindoline A peptide which were also analyzed in this work, are indicated in Table 1 [38,39,40,41,42]. In membrane-mimicking environments, Tritrp normally adopts a well-defined amphipathic two turn structure, where the two Pro residues are involved in the formation of the two turns in the peptide backbone [36]. Structural NMR and spectroscopic studies have shown that substitution of both Pro residues by Ala in Tritrp results in the creation of a mostly alpha helical backbone structure ([37,43], see also [44]). In a similar fashion, substitution of Pro residues by Ala in the related Trp-rich AMP indolicidin also led from a turn structure to a more alpha-helical structure [45,46]. Our results show that several Tritrp analogs possess good antibiofilm activities against *P. aeruginosa*. However, some of the more potent antibiofilm peptides that were uncovered also have high hemolytic activity against red blood cells [37] and can act as cytotoxic agents on peripheral blood mononuclear cells (PBMCs) as well [25], necessitating a careful selection of the most optimal peptides for potential clinical use.

## 2. Results

### 2.1. Antibiofilm Activities Against P. Aeruginosa

**Figure 1:**
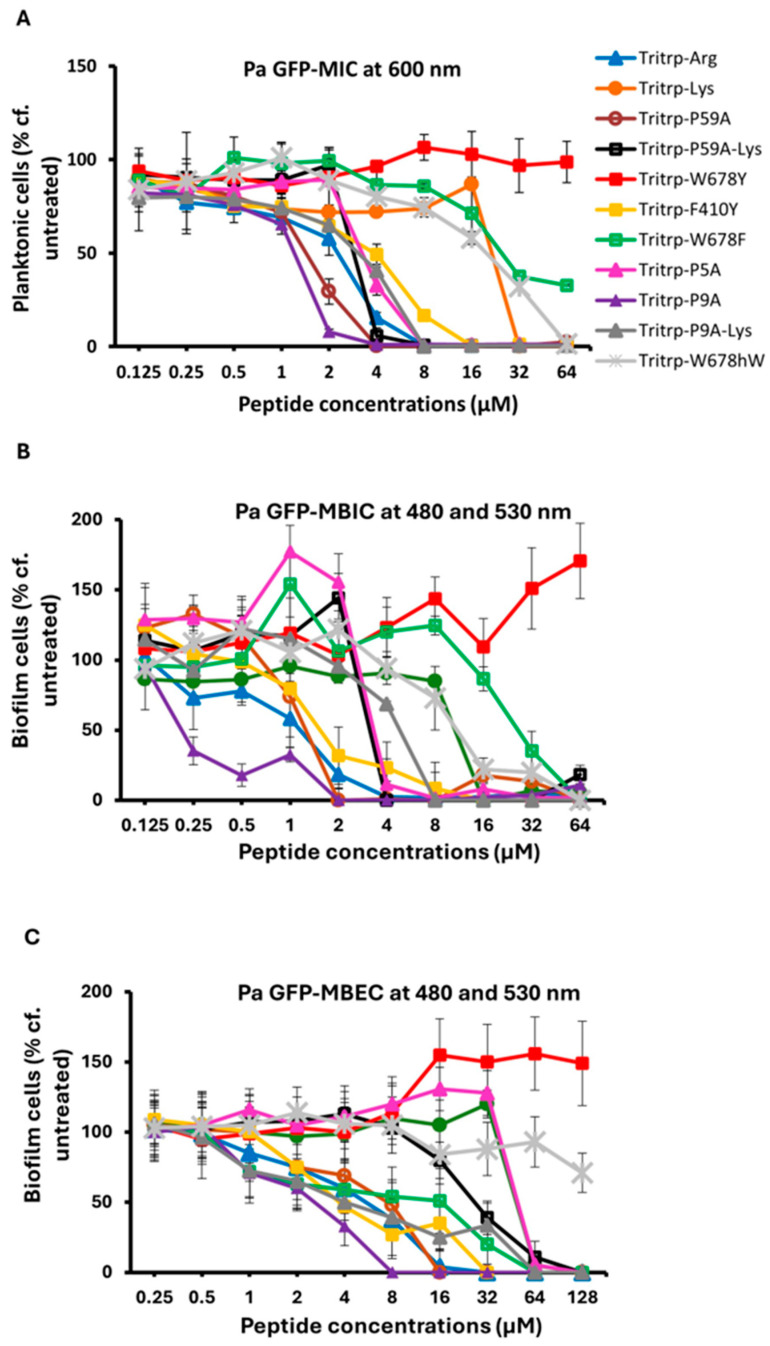
The MIC (**A**), MBIC (**B**) and MBEC (**C**) values for the tritrpticin analog peptides were assessed against Pa PAO1 GFP. The MIC values were determined by measuring the absorbance of bacterial cells at 600 nm. The MBIC and MBEC values were determined by recording the fluorescence at 480 nm and 530 nm from the biofilms of GFP-expressing PAO1. Three biological replicates were run for each test sample, and the mean was calculated for the recorded absorption or fluorescence values. In the graphs, the error bars represent the standard deviation (±SD).

The MIC, MBIC and MBEC activities that were measured for Tritrp and its Arg, Pro and aromatic amino acid-substituted analogs are shown in Figure 1 and they are tabulated in Table 2. For comparison, the activities measured for the related Trp-rich peptide indolicidin and two puroindolineA variants are also shown, as well as the activities of the two most potent hexapeptides that were evaluated in our previous study [23]. It is clear from these data that the original amidated Tritrp peptide with Arg residues (Tritrp-Arg) is more active in terms of its MIC, MBIC and MBEC than the one with Lys residues (Tritrp-Lys). Going down the list, we notice that in particular, two of the analogs with Pro substitutions have the most potent antibiofilm activities, Tritrp P59A with both Pro5 and Pro9 substitutions, or Tritrp P9A with only a Pro9 to Ala substitution. This trend is seen for the MIC, MBIC and MBEC values measured. The Tritrp analogs with all three Trp substituted by Tyr (Tritrp-W678Y) or with all Trp substituted by Phe (Tritrp-W678F) both show a poor activity compared to the parent peptide. This is likely related to the fact that the Tyr residues in the Tritrp-W678Y peptide generally remain more solvent exposed when bound to a membrane [47]. Moreover, since the phenyl ring of Phe likes to reside in the interior of the membrane [47], the Tritrp-W678F analog behaves quite differently from the natural variant, as the Trp indole rings normally prefer to reside in the membrane interface region [48,49,50]. On the other hand, the peptide Tritrp-F410Y with the two Phe residues of Tritrp substituted by Tyr behaves in a similar manner as the parent peptide. Comparisons of all the Tritrp analogs, with the two Trp- and Arg-rich hexapeptides and with the antimicrobial and antibiofilm values obtained for indolicidin and puroindolines, shows that several of the Tritrp analogs have the most potent antibiofilm MBIC and MBEC activities when acting on *P. aeruginosa* PAO1. They clearly improve on the performance of the hexapeptides and display slightly better activities than the related Trp-rich indolicidin and puroindoline AMPs.

**Figure 2:**
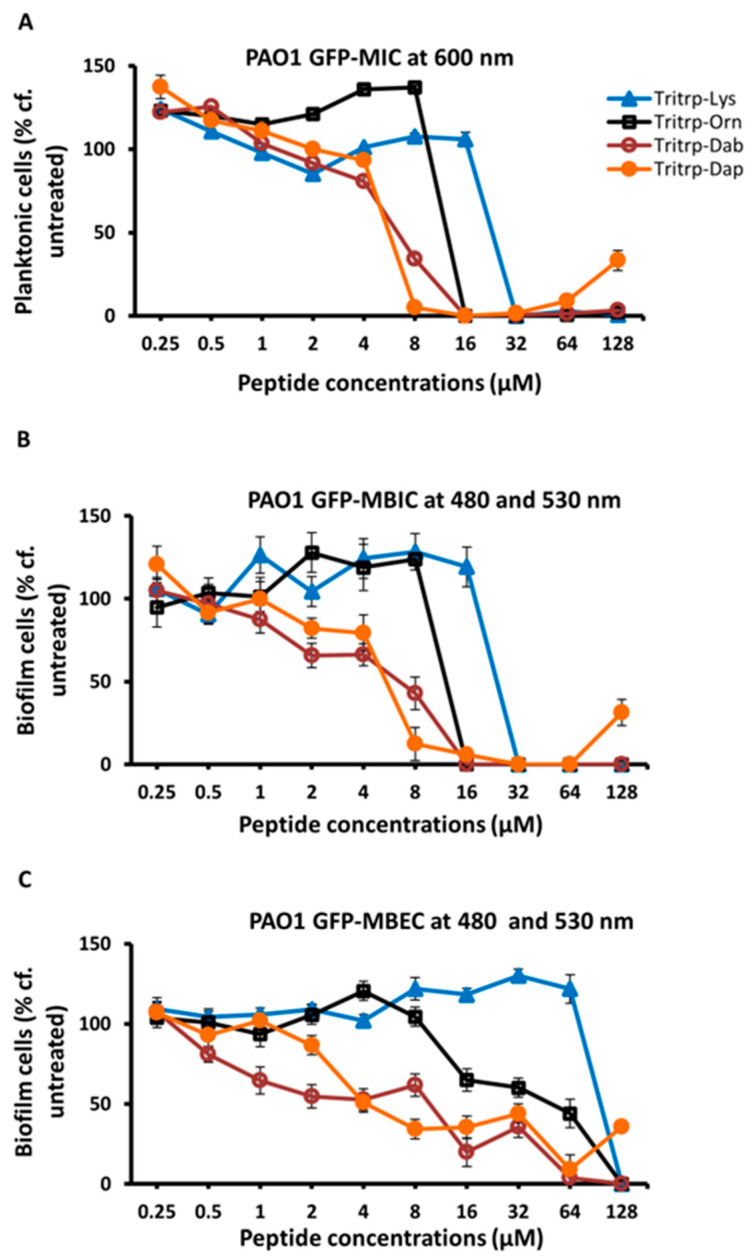
The MIC (**A**), MBIC (**B**) and MBEC (**C**) values for the tritrpticin-Lys derivative peptides were assessed against Pa PAO1 GFP. The MIC values were determined by measuring the absorbance of bacterial cells at 600 nm. The MBIC and MBEC values were determined by recording the fluorescence at 480 nm and 530 nm from the biofilms of GFP-expressing PAO1. Three replicates were run for each sample, the values were averaged and the error bars represent the standard deviation (±SD).

In a previous study [32], we found that substitutions of Arg and Lys in Tritrp with unnatural amino acids with shortened sidechains, such as ornithine (Orn), 2,4-diaminobutyric acid (Dab), or 2,3-diaminopropionic acid (Dap), which have three, two and one methylene groups, respectively, in the sidechain, could give rise to AMPs with improved antimicrobial potency. In a follow-up study, we also found that some of these analogs, in particular the shorter Lys versions, also provided better selectivity, and had reduced cytotoxic effects on RBCs and PBMCs [25]. Hence, we decided to evaluate the antibiofilm activities of these Lys variants of Tritrp. The results are shown in Table 3 and in Figure 2. These data show that the Dab- and Dap-substituted Tritrp variants containing the shortest sidechain variants of Lys also showed improved antibiofilm properties (MBIC and MBEC) when compared to the Lys version of the peptide.

### 2.2. Antibiofilm Activities Against S. Aureus

**Figure 3:**
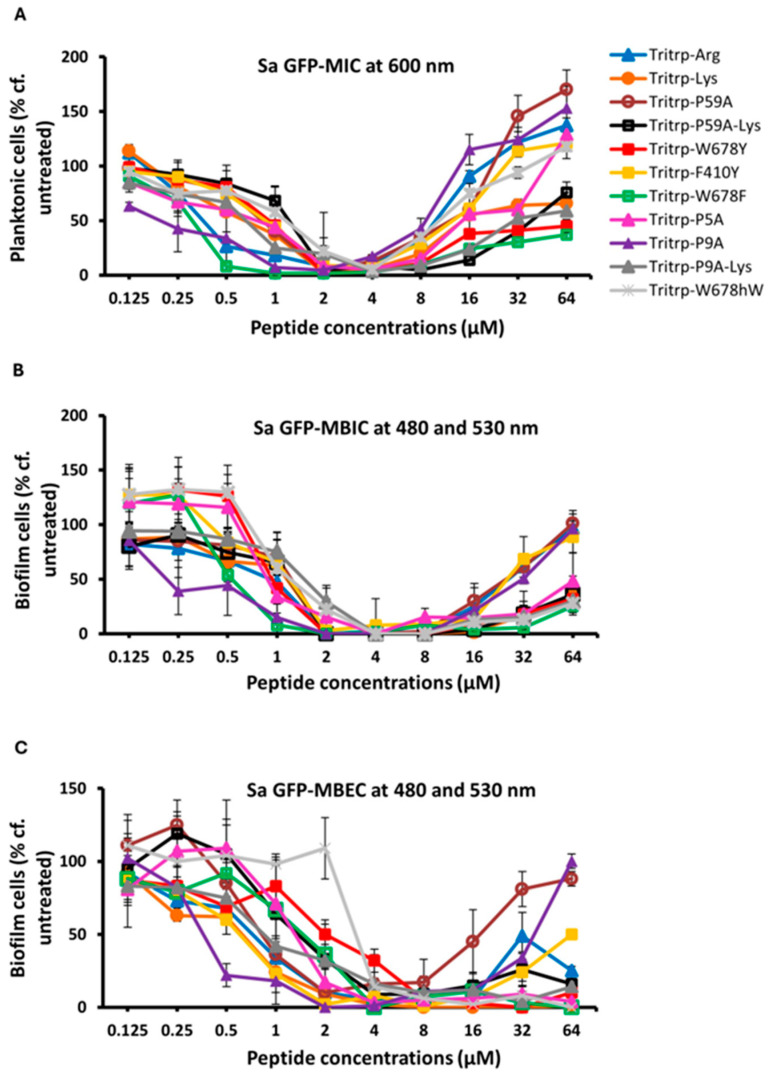
The MIC (**A**), MBIC (**B**) and MBEC (**C**) values for the tritrpticin analog peptides were assessed against Sa GFP. The MIC values were determined by measuring the absorbance of bacterial cells at 600 nm. The MBIC and MBEC values were determined by recording the fluorescence at 480 nm and 530 nm from the biofilms of GFP-expressing Sa. Three biological replicates were run for each test sample, and the mean was calculated for the recorded absorption or fluorescence values. In the graphs, the error bars represent the standard deviation (±SD).

In our previous studies [23], we found that the two Trp- and Arg-rich hexapeptides listed in Table 1 displayed excellent activities against a Gram-positive *S. aureus* MBEC strain. Hence, it was of interest to us to determine if this activity could be improved upon by utilizing the 13-residue Tritrp peptides for eradicating biofilms formed by this Gram-positive strain. Our data are shown in Figure 3 and in Table 4. Taken together, these data show that the Tritrp peptides do not offer a serious improvement in the MIC, MBIC and MBEC values over the two hexapeptides described earlier. Given that several of the Tritrp analogs have relatively high hemolytic activities [37], while the hemolytic activity of the shorter hexapeptides is negligible [51], the latter are clearly preferred for potential clinical applications against *S. aureus* and potentially for other Gram-positive strains.

### 2.3. Antibiofilm Activity Measured by Different Methods

**Figure 4:**
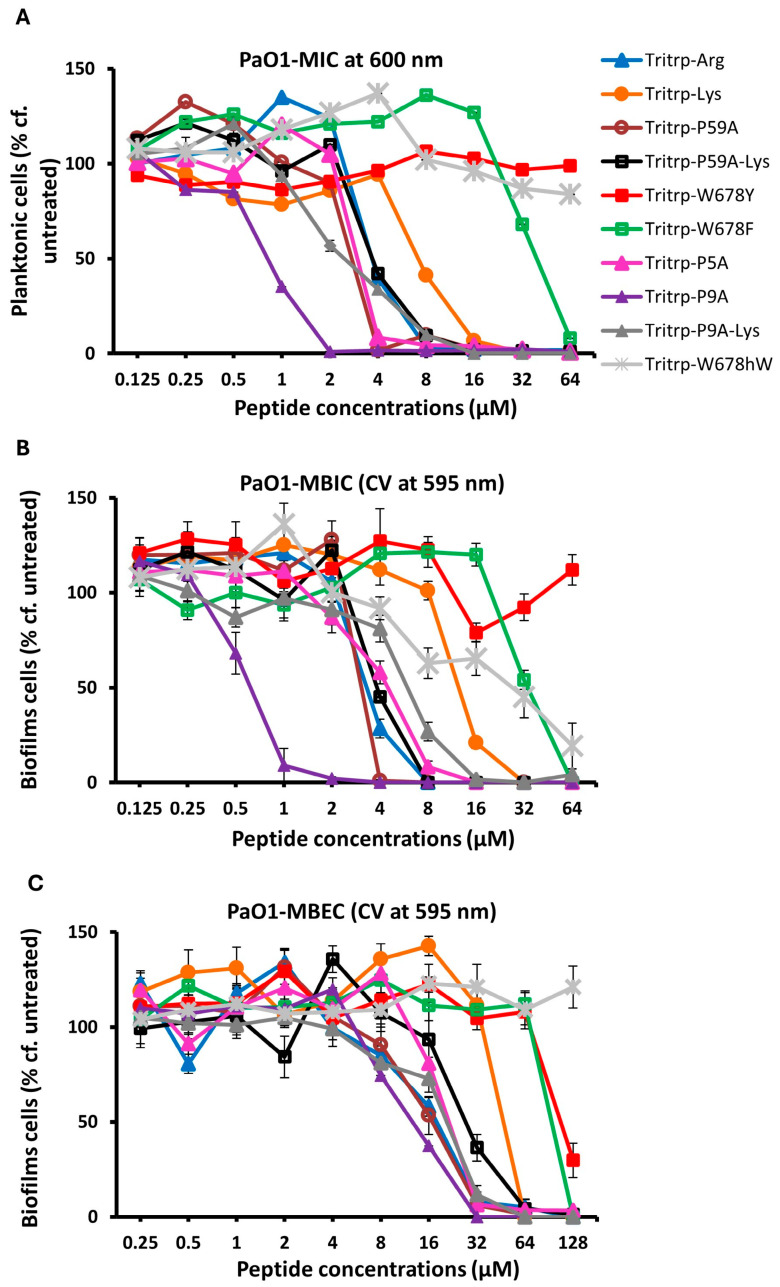
The MIC (**A**), MBIC (**B**) and MBRC (**C**) values for the peptides were assessed against *P. aeruginosa* PAO1 in the presence of 1× BM2. The inhibition of planktonic cells, the inhibition of biofilm cells and the reduction in biofilm cells were determined for the peptides by recording of the planktonic cells at 600 nm and quantification of biofilms formed by Pa PAO1 using crystal violet at 595 nm, respectively. Three biological replicates were run for each test sample, and the mean was calculated for the recorded absorption values. Data points with error bars indicate the standard deviation (±SD).

**Figure 5:**
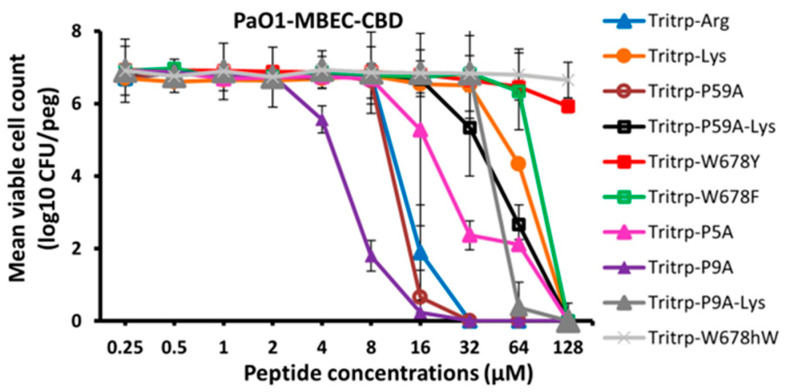
The MBEC values for the peptides were assessed against *P. aeruginosa* PAO1 in the presence of 1× BM2 medium. The eradication of biofilm cells was determined for the peptides using the counting of biofilm cells formed by Pa PAO1. Three biological replicates were run for each test sample, and the mean was calculated for counted cell values. Data points with error bars indicate the standard deviation (±SD).

In order to ascertain that the antibiofilm activities measured for the Tritrp analogs by the fluorescence method while using the GFP-producing *P. aeruginosa* strain were accurate, we also re-measured the MBIC and MBEC antibiofilm activities by the standard crystal violet assay [52], using a regular *P. aeruginosa* PAO1strain that does not produce the GFP protein and that we have used in an earlier study [22]. These results are depicted in Figure 4 and in Table 5. We note the close agreement with the MBIC and MBEC values determined by the fluorescence and CV methods, similar to what we found in our previous work for an *S. aureus* MRSA strain [23]. We also re-evaluated the MBEC values that are measured using the Calgary biofilm device (CBD), where biofilms form on pegs, rather than at the bottom of the 96-well plates [53]. Another feature of this CBD method is that the cell count is measured by plating out the cells from the pegs, making it a more stringent test; these results are depicted in Figure 5. While the exact numbers are not always the same, when compared to the results shown in Figure 2, the trends in Figure 5 are clearly identical to the earlier results, and we note again that two of the Pro-substituted analogs consistently showed the best MBIC and MBEC activities. In particular, we find that the single Pro9Ala Tritrp-P9A-substituted version has better MIC, MBIC and MBEC activities than the parent peptide as well as the Tritrp-P59A analog with both prolines substituted to Ala.

**Figure 6:**
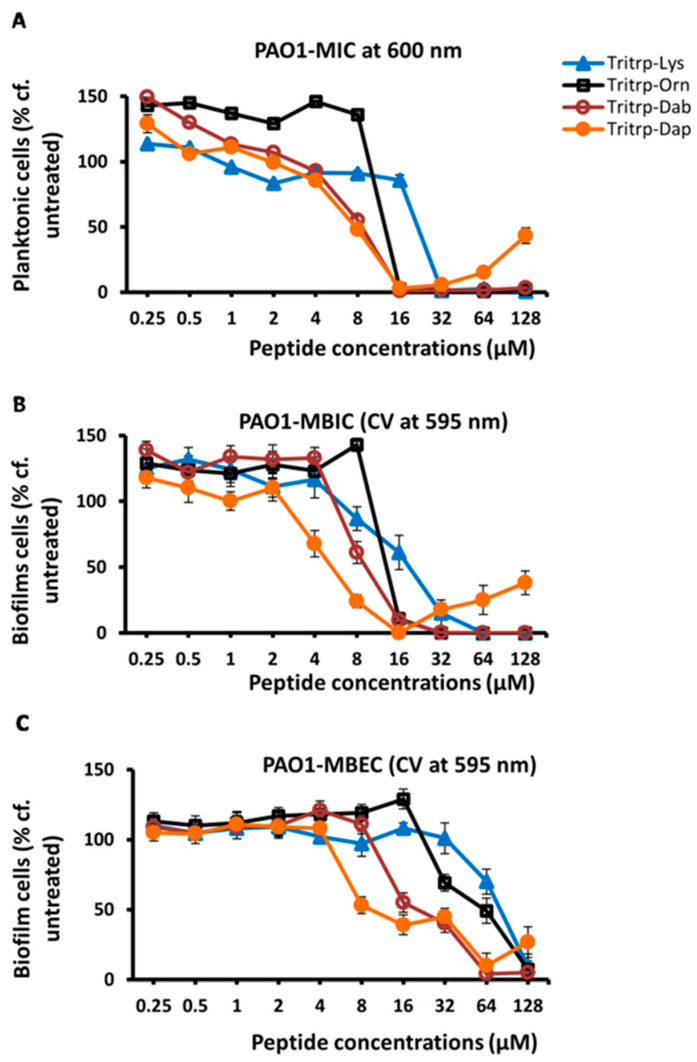
The MIC (**A**), MBIC (**B**) and MBEC (**C**) values for the tritrpticin-Lys derivatives against *P. aeruginosa* PAO1 were assessed by the crystal violet (CV) assay for the measurement of inhibition of biofilm formation and eradication of biofilm cells, respectively. The biofilm cells were rinsed, stained with 0.1% crystal violet for 15 min and then rinsed again. The CV was released from biofilm cells by the addition of 95% ethanol and quantified by reading on a spectrometer at 595 nm. Three replicates were run for each sample, the values were averaged and the error bars represent the standard deviation (±SD).

Finally, we also analyzed the antibiofilm activities of the Lys-substituted Tritrp peptides with the crystal violet method and the regular *P. aeruginosa* PAO1 strain. These results are depicted in Figure 6 and in Table 6. Comparing these data with those in Table 3 and Figure 2, we note again that there is an excellent agreement between the values that were measured with these two different methods to assess biofilm formation. As already indicated above, this concurrence was also noted in our previous study for *S. aureus* peptide treatments [23].

### 2.4. NMR Analysis of the Solution Behavior of Tritrpticin

As has been shown previously, the ^1^H NMR spectra of Tritrp are complex because in aqueous solution, the peptide exists with several slowly interconverting conformations. In our experience, this is most easily detected without serious peak overlap by studying the imino-protons around 10 ppm of the three Trp indole groups in the peptide [30,36,37]. This complexity disappeared when the peptide was bound to membrane mimetics, suggesting that a single conformer is found for the membrane-bound AMP. Similar results for Tritrp binding to various membrane mimetics have been reported by other researchers [54].

**Figure 7:**
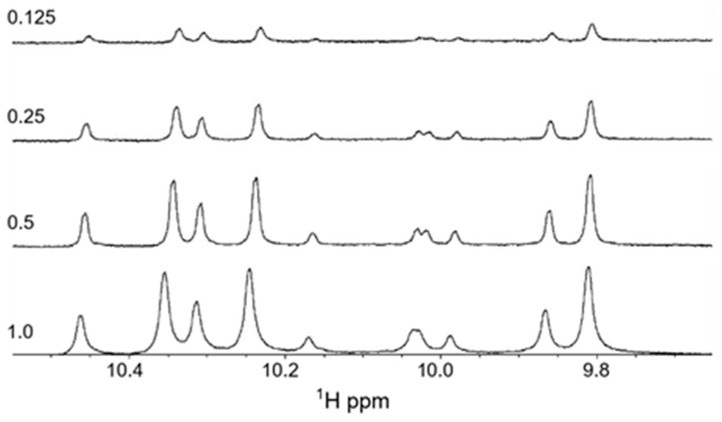
One-dimensional proton NMR spectra recorded for the tryptophan imino-proton region of tritrpticin in aqueous solution at 37 °C. The peptide concentrations (in mM) are indicated in the figure.

As tritrpticin is a Trp- and Arg-rich peptide there is the potential that the formation of cation–pi interactions, which are strongly favored to occur for Trp and Arg sidechains [55,56,57], could lead to forming dimeric or multimeric peptide species. Therefore, we wanted to investigate if such intermolecular interactions occurred and contributed to the spectral complexity. ^1^H NMR spectra were recorded at four different peptide concentrations (shown in Figure 7) to determine if there were any concentration-dependent changes in the spectral appearance. They all show complex spectra with multiple peaks, and it is noteworthy that the ratio of the peak intensities is maintained—as are the peak linewidths—suggesting that it is unlikely that such intermolecular complexes are formed. We also recorded ^1^H NMR spectra for the Lys-substituted form of the peptide, as Lys-Trp cation-pi complexes are highly unlikely to form in aqueous solution [56] These spectra showed similar multiple conformations and peak distributions as the Arg-containing peptide (for details see [37]), further supporting this notion.

**Figure 8:**
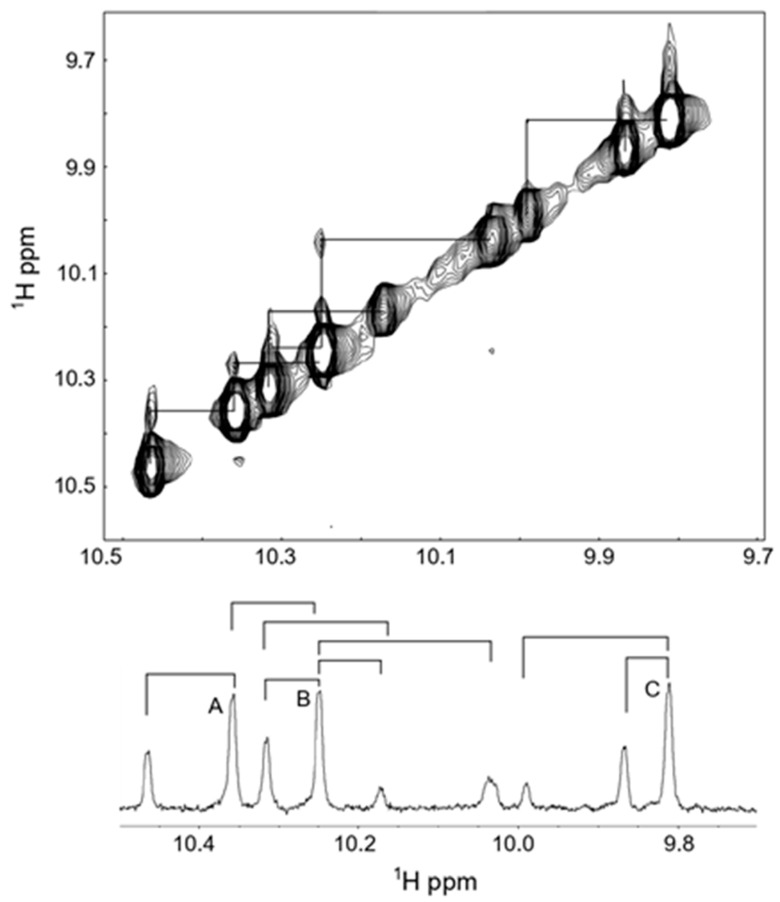
Two-dimensional ROESY spectrum of 1M tritrpticin-NH_2_ (Tritrp-Arg) were collected in aqueous solution at 37 °C. The imino-region of the spectrum is shown, and the observed exchange interactions are depicted below over the 1D spectrum. A, B and C refer to the three main peaks observed.

To study if some of the Trp imino-NH peaks showed exchange behavior, we subsequently recorded the 2D ROESY spectra (see Figure 8). These results clearly show that there are interconverting species, which are related to Pro cis–trans isomerism. As can be seen, the three most intense peaks (labeled A, B and C) all have a partner peak that is positioned downfield (by < 0.1 ppm) from these main peaks. Because these six peaks collapse into three when Pro9 is substituted by Ala [37], we can ascribe these to the trans and cis peptide backbone structures of the Trp-Pro-Phe bond, where about 70% is in the trans form and 30% is in the cis form. The ratio of cis to trans around a regular X-Pro bond in a linear peptide is normally between 6 and 10%, and hence we have a much higher ratio. The high percentage of the cis-bond is caused by the stabilizing influence of the Trp sidechain in the i − 1 position on the cis form, a notion that has been studied and demonstrated by several authors [58,59]. Indeed, in one interesting study, a cis/trans ratio of 40/60% was found for a Trp-Pro bond that is involved in the activity of a protein involved in the regulation of circadian rhythms [60]. In that case, the authors demonstrated that the imino protons were downfield-shifted in the cis form compared to the trans form, similar to what we see here. Substitution of Pro5 of Tritrp by Ala leads to the disappearance of all the smaller Trp-imino peaks in the spectrum [37], indicating that these arise from cis/trans isomerism around the Phe-Pro-Trp bond. Several of these smaller peaks are seen to be in exchange in the 2D ROESY spectrum, with the larger peaks demonstrating interconversions around the latter X-Pro bond. These peaks reflect about 7%, which is a more normal ratio. Although the large cis/trans ratios are frequently seen in linear peptides and in (intrinsically) unfolded proteins [61], the large difference in the cis/trans ratios for the two X-Pro bonds in Tritrp was a bit unexpected, as all aromatic residues (Phe, Tyr, Trp) are known to stabilize the cis X-Pro bond ([62,63,64], and references therein). Hence, it is unclear why the Phe-Pro-Trp in Tritrp does not give rise to a somewhat higher cis/trans ratio, but it is possible that other residues in the i + 1 position, and beyond, further modulate the cis/trans ratio [59].

## 3. Discussion

Several studies have shown that some AMPs possess antibiofilm properties, while others are devoid of such activities [11,14]. There are also examples of naturally occurring bioactive peptides, such as the atrial natriuretic peptide, that have no antimicrobial activities against planktonic cells, but that can interfere in bacterial biofilm formation (e.g., [65]). While the chemical features that make a potent AMP are now quite well understood (cationicity, hydrophobicity, amphipathic membrane-bound structure), there are currently no well-established design features for developing antibiofilm peptides. This is probably related to the fact that widely different mechanisms of action have been proposed that can achieve antibiofilm action [14,66,67]. Moreover, cells directly dispersed from biofilms seem to have unique properties when compared to normal planktonic cells, providing further complexity [68]. Hence, the screening of AMPs for antibiofilm properties, such as performed in this study, is currently the best way to uncover new antibiofilm peptides. For example, through a screening approach, we recently identified that short Trp- and Arg-rich hexapeptides are very potent at eliminating biofilms from an MRSA strain of *S. aureus* [23]. These peptides with only six amino acids residues were much shorter than the twelve to thirty residues that are normally cited as being required to form an antibiofilm peptide [14,20]. While theoretical QSAR studies [69] have had some success in uncovering some new 12-residue peptides, this approach could not identify the chemical or structural features that characterize a potent antibiofilm peptide. Interestingly, one of the most potent QSAR-predicted peptides was quite rich in Trp and Arg residues [69]. However, it lacked the Pro residues and the proximal Trp residues that characterize indolicidin and tritrpticin.

In studies with several peptides, it has been observed that some AMPs can display antibiofilm activities at concentrations that are lower than their MIC values, which represents the concentration of an AMP required to kill planktonic cells (see, for example, [11,70]). Indeed, in their early studies, Overhage et al. demonstrated that LL37 and indolicidin both displayed such antibiofilm activities, while several other known AMPs did not show any activity, revealing that such effects are highly peptide-specific. Also, in our current work, we see such concentration effects, for example, comparison of the MIC and MBIC’s curves for Tritrp-P9A in Figure 1 and Figure 4 show that the latter curve is shifted to lower peptide concentrations. Likewise, in Figure 2, the shape of the curve for the MBEC suggests that more than half of the cells are already removed at a concentration of 4 uMol/L, which is below the MIC value. However, removing the remaining cells requires substantially higher peptide concentrations, suggesting that not all biofilm-entrapped bacteria in our MBEC assays are equally accessible to the AMPs.

In previous studies, it has been shown that the Trp-rich AMPs indolicidin and puroindolineA can display antibiofilm activities against several pathogenic bacteria [11,42]. In view of their comparable Trp-rich amino acid sequences, both these peptides are related to Tritrp, and hence we expected that the latter would display antibiofilm properties as well. Indeed, our assay results (summarized in Table 2 and Table 5) support this presumption, and they show that of these three Trp-rich peptides that are of equal length, Tritrp has the most promising antibiofilm activities. Interestingly, we find that there is a strong correlation between the MIC values on the one hand and the MBIC and MBEC activities on the other hand, suggesting that the underlying antimicrobial properties of Tritrp are at least partially responsible for the antibiofilm activity. It should be noted that this is not seen for all antibiofilm peptides previously studied [11,21,70], but the same trend was also found in our previous study of Trp- and Arg-rich hexapeptides [23]. Moreover, our subsequent analysis of a range of Tritrp analogs showed that the antimicrobial MIC values for these peptides were also related to their MBIC and MBEC values (vide supra). In further agreement with this notion, Talukdar et al. (2021) [42] reported that the Trp-rich purindolineB peptide had no antibiofilm activity against *Campylobacter jejuni*, which correlates with the fact that this peptide also did not show antimicrobial activities against *Escherichia coli* and *S. aureus* [41].

Our results also show that Tritrp clearly outperformed the two WWW hexapeptides when acting on *P. aeruginosa* PAO1 strains (see Table 2 and Table 5). Because of this potential, it was of interest to us to study several Tritrp analogs. It was found that substituting Trp by the other aromatic residues Phe or Tyr drastically reduced the antibiofilm activities. However, substituting both Pro residues simultaneously with Ala led to a more potent peptide; this may be related to the fact that this peptide acquires a more helical backbone structure when bound to membrane mimetics [37] in comparison to the native peptide which forms a well-defined amphipathic two turn structure [36]. Interestingly, the most potent antibiofilm (and antimicrobial) peptide was the one with a single Pro9Ala substitution (see Table 2 and Table 5). In its membrane-bound state, this peptide has a more extended backbone structure [37]. On the other hand, a single substitution of Pro5 with Ala did not have a comparable effect. Taken together, these results highlight the unique importance of the two Pro residues for the Tritrp activities.

It is well known that aggregation in aqueous solution can occur for various AMPs and that this can markedly influence their biological activity (e.g., [71,72]). However, our NMR results obtained for the peptide in aqueous solution showed that peptide aggregation through intermolecular cation–pi interactions between Trp and Arg sidechains is unlikely to occur and that the peptide appears to be in a monomeric state in aqueous solution at a pH of 4.0. We note that the high cis/trans ratio observed for Tritrp in our NMR experiments for the Trp8-Pro9 bond could potentially play a role in the antibiofilm activity.

Overall, our work highlights the potential of Tritrp analogs in comparison to related Trp-rich AMPs as potent antibiofilm peptides that can interfere in biofilms produced by *P. aeruginosa* and potentially other Gram-negative pathogenic bacteria. New antibiofilm agents are welcome for this organism, as *P. aeruginosa* is well-known to have numerous mechanisms to elude the action of antibiotics during biofilm formation [73]. Various factors can influence the suitability of AMPs to act as bactericidal and antibiofilm peptides simultaneously (for a general discussion see, for example, [74]). One key factor to consider with a view to future clinical applications is the hemolytic activity of the AMPs, as well as their activity against PBMCs; obviously, an ideal AMP should not be detrimental to the host. Unfortunately, the Tritrp P59A peptide has relatively strong hemolytic activity, while the Tritrp P9A peptide has a slightly more modest hemolytic activity that is comparable to indolicidin and somewhat better than the amidated Tritrp parent peptide [37]. The cytotoxicity activities of Tritrp P59A and Tritrp P9A against PBMCs are unfortunately also quite strong, but these can be improved upon by substituting Lys for the four Arg residues of Tritrp [25]. The notion that Arg- and Trp-containing peptides are more cytotoxic than the commensurate Lys peptides has also been reported previously by other authors and has been observed both in in vitro and in animal model studies (see, for example, [75,76]). While the antimicrobial and antibiofilm activity of such Lys-substituted peptides is typically somewhat reduced compared to Arg-containing peptides (see Table 2 and Table 4), their better selectivity for bacterial over human cells would make them the preferred choice in clinical settings. Shortening the length of the Lys sidechain [29,32,77] could further improve the selectivity [25], although these changes would need to be balanced by maintaining the antibiofilm activity. The data depicted in Figs 2 and 6 clearly indicate that substituting Lys with the shorter Dab or Dap unnatural amino acids leads to improved antimicrobial and antibiofilm activities. It is important to stress here that Tritrp-Dap has no effect on RBCs or PBMC’s [25], which would make AMPs substituted with this analog very suitable for clinical applications. While the sidechain amino groups of Orn and Dab have pKa values comparable to Lys, once incorporated into a peptide, Dap is an unusual amino acid with a sidechain pKa value of 6.3 which can perturb the backbone structure of the peptide [78,79]. Of note, shortening of the Lys sidechains also makes the peptides resistant to trypsin and other related proteolytic activities [32,80,81], which could further enhance their activities in in vivo situations.

The antimicrobial mechanism of action of Tritrp has been investigated in some detail. Biophysical studies with vesicles made of model bacterial and mammalian membranes were evaluated by calcein leakage experiments and lipid flip–flop experiments. These studies indicated that Tritrp could act preferentially on the bacterial cytoplasmic membrane, likely forming dynamic toroidal pores that cause membrane leakage [30,37,82,83]. Also, calorimetry studies indicated that Tritrp could bind strongly to vesicles made of *E. coli* polar lipid extract and to other model membranes containing phospholipids with the negatively charged phosphatidylglycerol headgroups [82]. The latter are abundant in bacterial membranes, but they are absent in mammalian membranes [84]. In doing so, it can seriously perturb the regular bilayer structure of such membranes [85]. Additional studies with a living engineered *E. coli* strain showed that Tritrp and several of its analogs could perturb the inner membrane of this Gram-negative bacterium [30,32]. However, subsequent studies have also indicated the possibility of intracellular actions of Tritrp, where it could bind to DNA and interfere in intracellular macromolecular synthesis [31]. Tritrp and its analogs could enter the bacterial cell through disproportionation of transient toroidal pores [86]. In a similar manner, it has been shown that the puroindoline A peptide can also cause leakage in the bacterial cytoplasmic membrane, while causing perturbations of intracellular activities as well [41,42]. Likewise, indolicidin can also perturb cytoplasmic bacterial membranes [87] and it can inhibit the intracellular synthesis of DNA, RNA and proteins [45]. Recent studies with indolicidin indicate that at concentrations below the MIC, this peptide can induce extensive lipid flip–flop in bacterial membrane [88,89]. Taken together, these data indicate that Tritrp, indolicidin and puroindoline A share several mechanisms through which they can kill bacterial cells. By being able to form membrane pores, they are clearly distinct from the Trp- and Arg-rich hexapeptides, which are too short to form such pores.

In our recent study on the effects of the hexapeptides on *S. aureus* [23], we argued that the cationic Trp- and Arg-rich hexapeptides can cause unmixing of the bacterial cytoplasmic membrane by clustering with the headgroups of phosphatidylglycerol phospholipids, leaving behind domains enriched in phosphatidylethanolamine. By itself, the latter phospholipid does not form stable bilayers, and this could contribute to the bacterial membrane instability and to antibiofilm action [84,90]. The WWW motif of the hexapeptides was shown to be important to this action, and since Tritrp also carries this motif, it is possible that some membrane unmixing occurs here as well although the cytoplasmic membrane composition in Gram-negative strains is somewhat different than in Gram-positive strains [84]. Moreover, as discussed above, since longer antibiofilm peptides are often active as antibiofilm agents at concentration below the MIC, it is tempting to speculate that the enhanced lipid flip–flop seen in the recent studies [88,89] also plays a role in the antibiofilm action against *P. aeruginosa*. Indeed, it has been shown that Tritrp, like indolicidin, can also cause lipid flip–flop for model bacterial membranes [37]. Finally, as Tritrp, indolicidin and puroindoline A can enter bacterial cells, where they have been shown to disturb macromolecular DNA, RNA and protein synthesis, these peptides could also bind to intracellular regulatory nucleotides, such as (p)ppGpp. Another well-studied antibiofilm peptide (IDR-1018), can bind to (p)ppGpp, and has been shown to also give rise to antibiofilm dispersal activities at low peptide concentrations [91]. While this peptide may exert other effects in the cell as well [92], (p)ppGpp is known to play an important role in bacterial antimicrobial resistance [93]. Antibiofilm peptides have also been shown to bind to the cyclic-di-GMP regulatory nucleotide that is known to play a role in biofilm formation [94,95]. Nonetheless, all these intracellular actions require the AMP to first cross the bacterial cytoplasmic membrane, and hence membrane interaction of the peptide with bacteria in biofilms must play a pivotal role in such activities. Finally, some researchers have suggested that these peptides seem to behave like ‘dirty drugs’ that lack specificity and can act on various targets simultaneously [10]. As such, it seems likely that several of these different mechanisms could all play together, perhaps sequentially, and it is therefore difficult at this time to pinpoint a unique antibiofilm target and a specific mode of action.

Based on the results reported herein, taken together with our previous study [23], we can conclude that Trp- and Arg-rich hexamer peptides appear to be the best way forward to try to address biofilm formation by Gram-positive organisms, while abbreviated-Lys and Pro-substituted versions of Tritrp offer the best path forward towards tackling Gram-negative bacteria. In combatting biofilms comprised of mixed bacterial communities that contain both Gram-negative and Gram-positive strains, which occur quite frequently [96], the Tritrp peptides seem to offer the most optimal choice, as these are equally effective (see Table 4) at killing Gram-positive *S. aureus* strains when compared to the hexapeptides. Be that as it may, this screening study is only a first step towards the development of antibiofilm agents that may be of clinical use. While the biofilm matrices studied in our work would certainly contain extracellular DNA that originates from the bacteria [97,98], biofilm matrices found in human hosts also contain DNA from the host which seems to provide a coating that surrounds the bacterial biofilm [99]. It is well known that many cationic AMPs have the propensity to bind to DNA (e.g., [100]). Hence, such a human host DNA coat around the biofilm could prevent an AMP from successfully penetrating such biofilms, which would render them inactive. Clearly, future studies will have to focus on measuring the antibiofilm efficacy of AMPs in wounds and skin infections or in animal models of infectious diseases [15], to provide better insights into their potential clinical effectiveness.

## 4. Materials and Methods

All peptides were chemically synthesized by standard Fmoc methods and were obtained from different sources, as previously described [25,37]. They were determined to be more than 96% pure by HPLC, NMR spectroscopy and mass spectrometry. All other laboratory chemicals used were obtained from VWR or Fisher-Scientific Canada. The flat bottom 96-well plates were obtained from Thermo Fisher Scientific.

The methods to determine the MIC, MBIC and MBEC have been described in detail in our previous publication [23]. The green fluorescent protein (GFP)-producing *P. aeruginosa* and *S. aureus* strains that were used in this work were also described in the earlier publication. Fluorescence detection was used as the main method to estimate the number of cells entrapped in the biofilm matrix. The methodology used for evaluation of the antibiofilm properties against an original *Pseudomonas* PA01 strain by crystal violet dye staining and the use of the Calgary biofilm device has been described elsewhere [22]. The regular PA01 strain was provided by Dr. J. Harrison, Calgary. All measurements were carried out on an Eppendorf AF2200 plate reader. Every 96-well plate included controls for bacterial cell growth without peptide treatment and media alone. All measurements were carried out in triplicate, and the values in the figures represent the average and the error bars are standard deviation.

As previously, all ^1^H NMR spectra of the peptide in aqueous solution (90% H_2_O/10% D_2_O) were collected at pH values close to 4.0 [30,37]. Spectra collected at pH values close to 7.0 showed the same features, but the peaks were much broader, which interfered with the analysis. A Bruker Avance spectrometer, operating at a frequency of 600 MHz, and equipped with a gradient triple resonance probe was used throughout. The two-dimensional (2D) ROESY spectra were collected with the standard Bruker pulse program and a mixing time of 400 msecs (1024 scans). ROESY spectra provide both NOE and exchange peaks that appear as cross peaks with a different phase (represented by different colors in the spectrum), and only the latter cross peaks were analyzed. As some exchange cross peaks were weak, they were confirmed by running the ROESY spectra multiple times.

## Figures and Tables

**Table 1:** Amino acid sequences and charges of the different tryptophan-rich peptides studied.

Peptides	Modifications	Amino Acid Sequences	Net Charges
Hexapeptide1		RRRWWW-NH2	+4
Hexapeptide2		RRWWWR-NH2	+4
Tritrp-Arg	Amidation	VRRFPWWWPFLRR-**NH2**	+5
Tritrp-Lys	Arg → Lys	V**KK**FPWWWPFL**KK**-NH2	+5
Tritrp-P59A	Pro → Ala	VRRF**A**WWW**A**FLRR-NH2	+5
Tritrp-P59A-Lys	Pro → Ala	V**KK**F**A**WWW**A**FL**KK**-NH2	+5
Tritrp-W678Y	Trp → Tyr	VRRFP**YYY**PFLRR-NH2	+5
Tritrp-F410Y	Phe → Tyr	VRR**Y**PWWWP**Y**LRR-NH2	+5
Tritrp-W678F	Trp → Phe	VRRFP**FFF**PFLRR-NH2	+5
Tritrp-P5A	Pro → Ala	VRRF**A**WWWPFLRR-NH2	+5
Tritrp-P9A	Pro → Ala	VRRFPWWW**A**FLRR-NH2	+5
Tritrp-P9A-Lys	Pro → Ala	V**KK**FPWWW**A**FL**KK**-NH2	+5
Tritrp-W678hW	Hydroxy-Trp	VRRFP(**hW**)(**hW**)(**hW**)PFLRR-**NH2**	+5
Indolicidin		ILPWKWPWWPWRR-NH2	+4
PuroA-Arg		FPVTW**R**WW**R**WW**R**G-NH2	+4
PuroA-Lys	Arg → Lys	FPVTW**K**WW**K**WW**K**G-NH2	+4

Bold letters indicate the substitutions in the amino acid sequence.

**Table 2:** The activities measured by the fluorescence method for the different tryptophan-rich peptides against *P. aeruginosa* PAO1 GFP.

Peptides (uMol/L)	PAO1 GFP (BM2)					
	MIC at 600 nm		MBIC at 488 and 530 nm		MBEC at 488 and 530 nm	
	LD50	LD90	LD50	LD90	LD50	LD90
RRRWWW-NH2	32	128	16	128	64	>128
RRWWWR-NH2	64	128	16	128	128	>128
Tritrp-Arg	4	8	2	8	8	16
Tritrp-Lys	32	32	16	32	64	64
Tritrp-P59A	2	4	2	4	8	16
Tritrp-P59A-Lys	4	4	4	4	32	128
Tritrp-W678Y	>64	>64	>64	>64	>128	>128
Tritrp-F410Y	8	16	2	8	4	32
Tritrp-W678F	32	>64	32	64	32	64
Tritrp-P5A	4	8	4	8	64	64
Tritrp-P9A	2	2	0.25	2	4	8
Tritrp-P9A-Lys	4	8	8	8	4	64
Tritrp-W678hW	32	64	16	64	>128	>128
Indolicidin	8	32	8	32	32	>128
PuroA-Arg	8	16	8	8	32	128
PuroA-Lys	4	8	4	8	64	64

Note: MIC, minimum inhibition concentration; MBIC, minimum biofilm inhibitory concentration; MBEC, minimum biofilm eradication concentration.

**Table 3:** The activities measured by the fluorescence method for the Tritrp-Lys derivatives against *P. aeruginosa* PAO1 GFP.

Peptides (uMol/L)	PAO1 GFP (BM2)					
	MIC at 600 nm		MBIC at 488 and 530 nm		MBEC at 488 and 530 nm	
	LD50	LD90	LD50	LD90	LD50	LD90
Tritrp-Lys	32	32	32	32	128	128
Tritrp-Orn	16	16	16	16	64	128
Tritrp-Dab	8	16	8	16	16	64
Tritrp-Dap	8	8	8	16	8	64

Note: MIC, minimum inhibition concentration; MBIC, minimum biofilm inhibitory concentration; MBEC, minimum biofilm eradication concentration.

**Table 4:** The activities measured by the fluorescence method for different tryptophan-rich peptides against *S. aureus* GFP.

Peptides (uMol/L)	Sa GFP (10% TSB and 0.1% Glucose)						Sa GFP
	MIC at 600 nm		MBIC at 488 and 530 nm		MBEC at 488 and 530 nm		MBC Agar Plate
	LD50	LD90	LD50	LD90	LD50	LD90	
RRRWWW-NH2	1	1	1	1	0.5	4	4
RRWWWR-NH2	1	1	1	1	1	4	4
Tritrp-Arg	0.5	2	1	2	1	2	2
Tritrp-Lys	1	2	2	2	1	2	4
Tritrp-P59A	1	2	2	2	1	2	4
Tritrp-P59A-Lys	2	2	2	2	2	4	4
Tritrp-W678Y	1	2	1	2	2	8	16
Tritrp-F410Y	1	2	2	2	1	2	4
Tritrp-W678F	0.5	0.5	1	1	2	4	8
Tritrp-P5A	1	2	1	4	2	4	4
Tritrp-P9A	0.25	1	0.25	2	0.5	2	2
Tritrp-P9A-Lys	1	4	2	4	1	8	8
Tritrp-W678hW	2	4	2	4	4	8	16
Indolicidin	4	8	4	8	2	8	8
PuroA-Arg	4	4	4	4	8	16	8
PuroA-Lys	2	4	2	4	8	8	8

Note: MIC, minimum inhibition concentration; MBC, minimum bactericidal concentration; MBIC, minimum biofilm inhibitory concentration; MBEC, minimum biofilm eradication concentration. The MBC was defined as the lowest concentration of peptide with no bacterial growth seen on the spot agar plate.

**Table 5:** The activities measured with the crystal violet method for tritrpticin analogs against *P. aeruginosa* PAO1.

Peptides (uMol/L)	PAO1 (BM2)							
	MIC at 600 nm		MBIC (CV at 595 nm)		MBRC (CV at 595 nm)		MBEC from Calgary Biofilm Device (CBD)	
	LD50	LD90	LD50	LD90	LD50	LD90	LD50	LD90
Tritrp-Arg	4	8	4	8	32	32	16	32
Tritrp-Lys	32	32	32	32	64	64	128	128
Tritrp-P59A	2	4	4	4	32	32	16	32
Tritrp-P59A-Lys	4	8	4	8	64	64	64	128
Tritrp-W678Y	>64	>64	>64	>64	128	>128	>128	>128
Tritrp-W678F	32	>64	64	>64	128	>128	128	128
Tritrp-P5A	4	8	8	8	32	32	32	128
Tritrp-P9A	2	2	1	2	16	32	8	16
Tritrp-P9A-Lys	4	8	8	16	32	64	64	64
Tritrp-W678hW	>64	>64	32	>64	>128	>128	>128	>128
Indolicidin	16	16	32	32	64	64	128	128
PuroA-Arg	8	16	16	16	32	32	16	64
PuroA-Lys	8	16	8	16	32	32	32	64

Note: MIC, minimum inhibition concentration; MBIC, minimum biofilm inhibitory concentration; MBRC: minimum biofilm reduction concentration; the MBEC (Calgary biofilm device (CBD)) was defined as the lowest concentration of peptide with no bacterial growth seen on the spot agar plate.

**Table 6:** The activities measured by the crystal violet staining method for the Tritrp-Lys derivatives against *P. aeruginosa* PAO1.

	PAO1 (BM2)					
	MIC at 600 nm		MBIC (CV at 595 nm)		MBEC (CV at 595 nm)	
	LD50	LD90	LD50	LD90	LD50	LD90
Tritrp-Lys	32	32	32	32	128	128
TriTrp-Orn	16	16	16	32	64	128
Tritrp-Dab	16	16	16	16	32	64
Tritrp-Dap	8	16	8	16	16	64

Note: MIC: minimum inhibitory concentration; MBIC, minimum biofilm inhibitory concentration; MBEC, minimum biofilm eradication concentration.

## Data Availability

Data are contained within the article. Further inquiries can be directed to the corresponding author.
